# Salivary Proteomics for Detecting Novel Biomarkers of Periodontitis: A Systematic Review

**DOI:** 10.1111/jre.13357

**Published:** 2024-12-02

**Authors:** Matteo Corana, Giacomo Baima, Giovanni Iaderosa, Francesco Franco, Jianjian Zhang, Giovanni Nicolao Berta, Federica Romano, Mario Aimetti

**Affiliations:** ^1^ Department of Surgical Sciences, C.I.R. Dental School University of Turin Turin Italy; ^2^ Department of Clinical and Biological Sciences University of Turin Turin Italy; ^3^ Department of Molecular Biotechnology and Health Sciences University of Turin Turin Italy

**Keywords:** biomarkers, diagnosis, inflammation, periodontal diseases, proteins

## Abstract

**Aim:**

Salivary content is regarded as a powerful diagnostic window for oral and systemic diseases and the proteomic profile could be useful to distinguish between different periodontal conditions. The aim of the present systematic review was to assess distinctive salivary proteins identified through untargeted proteomics in periodontitis patients compared to periodontally healthy and gingivitis subjects, as well as to provide a qualitative methodological assessment of the current literature.

**Methods:**

Relevant studies identified from Medline via PubMed, Scopus, Embase, and Cochrane Library databases were retrieved to answer the following PECO question: “In systemically healthy individuals, are there any differences in salivary protein expression profiles assessed in proteomics studies between patients with periodontitis and periodontally healthy or gingivitis subjects?” Moreover, diagnostic utility of the identified markers was sought via a targeted literature search and further quantitative assessment. A modified version of the QUADAOMICS tool was used for the quality assessment of the included studies.

**Results:**

After screening 461 relevant articles, a total of 13 studies were selected. The number of identified discriminant salivary proteins ranged from 2 to 4161. However, it was possible to identify proteins that were consistently over‐ or under‐expressed in periodontitis patients in at least 3 studies. Among these, complement C3, profilin‐1, SA100A8, and fibrinogen were consistently reported as increased in periodontitis, while cystatin‐SN and leukocyte elastase inhibitor were more elevated in periodontally healthy controls. Only 4 studies reported diagnostic accuracy measures, with SA100A8 showing an area under the curve of 0.71 (95% CI: 0.66–0.75) in meta‐analysis.

**Conclusions:**

Untargeted proteomics techniques identified some key biological molecules which were consistently reported to be over‐ or under‐expressed in periodontitis. These findings could be useful to support novel candidate biomarkers for periodontitis. The high level of heterogeneity in methods and reporting urge to develop standardized protocols to be implemented in this research field (PROSPERO CRD42022299826).

## Graphical abstract

Untargeted salivary proteomics identified promising biological molecules consistently over‐ or under‐expressed in periodontitis patients compared to periodontally healthy subjects, but further targeted approaches are required for their validation.

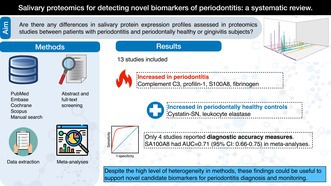

**Summary**

- Background
  ○ Salivary proteins profile can be regarded as resilient fingerprint to identify biomarkers useful for diagnosis and monitoring of periodontal diseases.
- Added value of this study
  ○ Despite heterogeneity in methods and reporting, the present systematic review identified relevant salivary molecules consistently over‐ (complement C3, profilin‐1, S100A8, and fibrinogen) and under‐ (cystatin‐SN and leukocyte elastase inhibitor) expressed in periodontitis patients compared to periodontally healthy subjects.
- Clinical implications
  ○ Consistent biomarkers identified through an untargeted proteomics approach could serve as potential candidates for future validation studies and may contribute to the development of diagnostic or prognostic tools in the future.

## 1 Introduction

Periodontitis is a highly prevalent chronic inflammatory disease that causes the irreversible destruction of tooth‐supporting tissues [1, 2]. Although the development of a dysbiotic pathogenic biofilm is the primary etiological factor, the onset and the progression of the disease are modulated by genetic, environmental, and behavioral factors [3, 4]. The diagnosis largely relies on clinical and radiographic assessments, although these diagnostic tools are more useful to assess past tissue destruction rather than disease activity and risk of progression [5]. Early identification of periodontitis would be key to preventing disease complications such as tooth loss and chewing impairment and to avoid more complex, costly, and less predictable treatments. Therefore, great efforts have been made in the search for sensitive molecular biomarkers to identify disease activity, to support early diagnosis, and to predict future progression at both patient and site level [6, 7, 8, 9].

Among oral fluids, gingival crevicular fluid (GCF) has been used as a potential diagnostic biological fluid because it contains site‐specific biomarkers of disease activity [10, 11]. It is regarded as an appropriate medium for local assessment of gingival inflammation and disease activity, but a full mouth collection is time consuming and technically demanding. Moreover, the small collection volume limits the opportunities of analysis using methods requiring larger quantities. Conversely, saliva is regarded as a convenient bio‐fluid to evaluate the entire‐mouth health state and a potential diagnostic tool for both systemic and periodontal diseases [12, 13, 14]. Saliva can be easily obtained with a minimal patient discomfort and contains a vast variety of biomarkers [15], including more than 5000 proteins from both human and microbial origin [16, 17]. Indeed, among human salivary proteins, approximately 73% appear to be unique of saliva, with the remaining 27% overlapping with serum proteins [15].

During the last decades, research interest has been directed toward the analysis of specific salivary molecules across different periodontal conditions, a pursuit largely enabled by the advancement of omics approaches [18, 19]. Indeed, by proteomic analysis, it is possible to systematically screen protein expression by both quantitative and qualitative means [20]. 2D Gel Electrophoresis is able to analyze complex protein mixtures by separating proteins first by their isoelectric point and then by their molecular weight. In several studies, 2D gel electrophoresis was used alone or coupled with mass spectrometry [21, 22]. However, approaches based on 2D gel are demanding, time‐consuming, and do not allow to test a large number of proteins [5]. High‐throughput proteomics method, such as protein microarrays, allowed to track a large number of proteins in parallel and to detect low abundant proteins, but the use of this technology is also restricted by the relatively high costs. To date, liquid chromatography coupled with mass spectrometry (LC–MS/MS) has been extensively applied to the study of periodontal diseases, focusing on microbial proteome, host proteome, or both [23]. This powerful analytical technique offers very high accuracy and selectivity by combining two different separative approaches based on analytes features (physico‐chemical properties with LC and mass‐to‐charge ratios with MS; [24]).

The present systematic review aimed at summarizing the available evidence regarding the salivary profile of patients with different periodontal conditions identified through untargeted proteomics studies, together with a methodological quality assessment of the included studies in order to evaluate risk of bias and propose quality improvements in methods and reporting. Moreover, the diagnostic accuracy of the most consistently identified biomarkers was further evaluated by an additional targeted literature search.

## 2 Methods

This research has been conducted in accordance with the Cochrane Handbook and reported according to the PRISMA guidelines [25, 26]. The protocol was registered on the International Prospective Register of Systematic Reviews (PROSPERO) under the number CRD42022299826.

### 2.1 Focused Questions

The present systematic review was designed to answer the following PECO question [27]:

In systemically healthy individuals, are there any differences in salivary protein expression profiles assessed in proteomics studies between patients with periodontitis and periodontally healthy or gingivitis subjects?

(P) Population. Adult patients in good systemic health.

(E) Exposure. Patients with a clinical diagnosis of periodontitis (P).

(C) Comparison. Subjects with healthy periodontal conditions (H) or gingivitis (G).

(O) Type of outcome measures. Differences in salivary protein expression profile or in single/combination protein markers between P and H/G. Additionally, the diagnostic accuracy of identified salivary protein biomarkers compared to gold standard clinical parameters was sought in both untargeted and targeted studies.

(S) Type of studies. Original studies in humans with observational design (cross‐sectional, case–control, and cohort) comparing periodontitis patients with periodontally healthy or gingivitis subjects in terms of salivary protein expression profile and/or concentration obtained through proteomics methods.

### 2.2 Eligibility Criteria

The target conditions were chronic (CP) and/or aggressive (AgP) forms of periodontitis according to the 1999 classification [28], or periodontitis of any stage, grade, and extent according to the current classification [29]. The diagnosis of periodontitis was based on clinical parameters (clinical attachment level and probing pocket depth) or a combination of clinical and radiographic parameters. Studies specifically involving less than 10 subjects per group, patients suffering from systemic diseases, as well as studies involving pregnant females were excluded. Furthermore, literature reviews, editorials, clinical case reports, animal studies, and in vitro experimental models were excluded, as well as articles not written in English.

### 2.3 Search Methods for the Identification of Studies

The literature search was performed in duplicate by two reviewers (MC and GI) who were calibrated for study screening, data extraction, and risk of bias assessment against another experienced reviewer (GB). The search was performed on four electronic databases [National Library of Medicine (Medline via PubMed), Scopus, Embase, and Cochrane Library] until October 2023 without any restriction on date of publication. Combinations of controlled terms (MeSH and EMTREE) and keywords were used as follows:

(“periodontitis” OR “periodontal disease” OR “periodont*”) AND (“MS” OR “MS/MS” OR “MS–MS” OR “LC/MS” OR “LC–MS/MS” OR “MALDI‐TOF” OR “MALDI‐TOF‐MS” OR “SELDI‐TOF” OR “SELDI‐TOF‐MS” OR “MALDI” OR “SELDI” OR “mass spectrometry” OR “liquid chromatography” OR “matrix‐assisted laser desorption/ionization” OR “surface‐enhanced laser desorption/ionization” OR “time‐of‐flight” OR “proteomics” OR “proteomic” OR “proteomic analysis”) AND (“saliva” OR “salivary”).

In addition, the references of all included studies and previous systematic reviews were cross‐checked by the same two reviewers (MC and GI).

### 2.4 Study Selection

Titles and abstracts of eligible studies were screened independently by two reviewers (MC and GI). Full text of studies that met the inclusion criteria were obtained for independent assessment by the same reviewers. Any disagreement was resolved by discussion or by consultation with a third reviewer (GB). The reasons for exclusion of studies after full text analysis were recorded. The inter‐reviewer reliability (percentage of agreement and kappa coefficient) of the screening method was calculated.

### 2.5 Data Extraction and Management

Data were independently extracted by two authors (MC and GI) using specifically designed templates. In case of a prospective clinical study, only baseline data (before any periodontal treatment) were extracted. In studies comparing periodontal status in patients with and without systemic diseases (such as diabetes), only data on systemically healthy individuals were extracted and analyzed.

### 2.6 Risk of Bias in the Included Studies and Quality Assessment

The included studies were analyzed in terms of risk of bias and quality assessment in duplicate by two reviewers (MC and GI) using a modified version of the NIH Quality Assessment Tool for Observational Cohort and Cross‐Sectional Studies and the QUADOMICS tool, specifically developed to evaluate omics research [30]. The tool comprises of 15 items about research question, study population, exposure, sampling procedures, confounding factors, outcomes, and statistical method. Every item was given a maximum of 1 point, and the total scores are interpreted as follows: 0–3, very low quality; 4–7, low quality; 8–11, moderate quality; and 12–15, high quality.

### 2.7 Pathway Enrichment Analysis and Strategy for Data Synthesis

We performed an enrichment analysis on proteins showing differential expression levels related to periodontitis using Flame tool (v2.0) (23). Outputs with a *p*‐value < 0.05 in the categories of gene ontology biological process (GO‐BP) and gene ontology cellular component (GO‐CC) were considered. Due to a high heterogeneity in methodology and outcomes among the included studies, meta‐analysis of data was not feasible. However, a consistency analysis was reported identifying protein markers significantly discriminant between P and G/H groups. Moreover, an additional literature search (Appendix 1) was performed evaluating the diagnostic ability of the identified markers. Data on area under the curve (AUC) receiver operating characteristic (ROC) curve, sensitivity, and specificity of the selected biomarker were extracted from the studies reporting them and meta‐analyses were sought. When quantitative synthesis was possible, statistical heterogeneity was tested with Cochran's *Q* test, and inconsistency evaluated as the *I* ^2^. Fixed effect model (inverse variance method) was used and forest plot was generated.

## 3 Results

### 3.1 Study Selection

**FIGURE 1:**
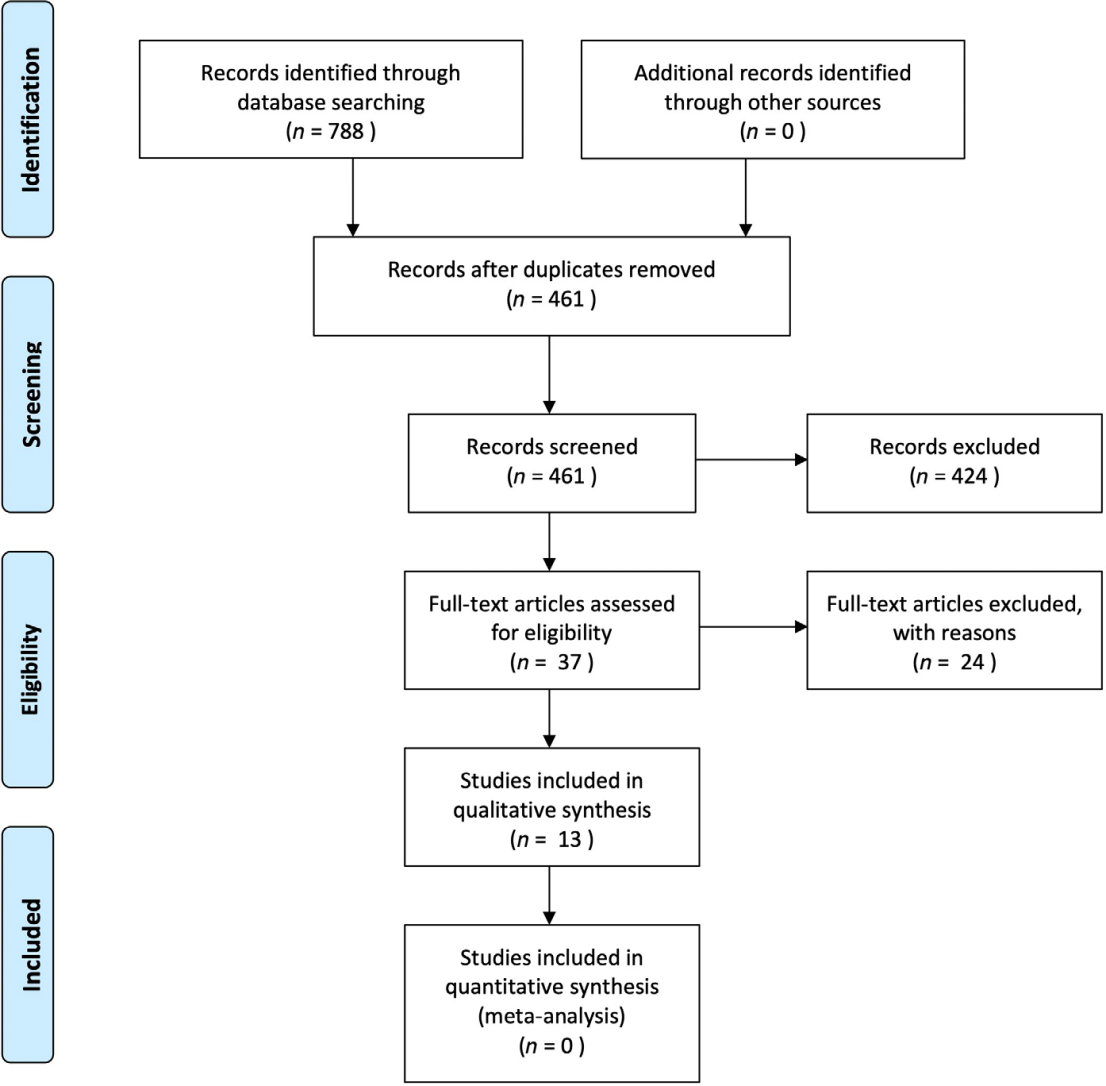
PRISMA flow‐chart of the study design.

Figure 1 depicts the study flow chart. In total, 461 titles were obtained from the electronic search. One additional document was identified from the manual search. After the screening of titles and abstracts, 37 articles were analyzed in full text. Among them, 24 papers were excluded for various reasons (summarized in Table S1). Finally, 13 articles were selected for the present systematic review [9, 31, 32, 33, 34, 35, 36, 37, 38, 39, 40, 41, 42, 43]. The measure of inter‐reviewer agreement was *k* = 0.86 for abstract screening and *k* = 0.91 for full‐text analysis.

### 3.2 Characteristics of the Included Studies

The characteristics of the included studies are summarized in Table 1. Six studies were conducted in Europe, 4 in Asia, and 3 in South America. The number of participants ranged from 20 to 141 (cases ranging from 10 to 67 and controls ranging from 10 to 74), with a mean age of participants ranging from 25 to 64 years. Eleven studies had a cross‐sectional design. The two remaining studies had a prospective design (also evaluating periodontitis groups before and after periodontal therapy). Periodontitis was defined according to different case definitions. Only one paper was focused on aggressive periodontitis. Two studies classified periodontitis patients in two groups (mild to moderate periodontitis/advanced periodontitis and chronic/aggressive periodontitis) and only three studies included patients with gingivitis. In most of the included studies, smoking was an exclusion criterion (8 out of 13).

**TABLE 1:** Characteristics of studies and participants in the systematic review.

Authors (year)	Region	Study design	Sample size (Males/Females)		Age (years; Mean ± SD)		Periodontal definition selected		Periodontal status at baseline		Periodontal status assessment
			Case	Control	Case	Control	Case	Control	Case (%)	Control (%)	Case
Gonçalves et al. (2010) [36]	Espírito Santo, Brazil	Cross‐sectional	10 (5/5)	10 (5/5)	45.0 ± 5.1	35.6 ± 9.5	CP: > 35 years of age, at least 6 sites on different teeth with BoP, PD ≥ 6 mm and CAL > 5 mm	H: BoP < 10% and PPD < 3 mm	10 (50.0)	10 (50.0)	Not reported
Salazar et al. (2013) [40]	Greifswald, Germany	Cross‐sectional	20 (10/10)	20 (10/10)	48.6 ± 11.4	49.6 ± 10.2	P: BoP > 10%, PD ≥ 5 mm at ≥ 2 sites and PD ≥ 4 mm at ≥ 40% of teeth	H: BoP < 30% and PD ≤ 3 mm	20 (50.0)	20 (50.0)	Partial‐mouth
Chaiyarit et al. (2015) [35]	Khon Kaen, Thailand	Cross‐sectional	30 (16/14)	30 (13/17)	51.2 ± 11.1	54.4 ± 11.0	CP: ≥ 10 remaining teeth with CAL > 5 mm and PD > 6 mm	H: no further specified	30 (50.0)	30 (50.0)	Not reported
Belstrøm et al. (2016) [32]	Copenhagen, Denmark	Cross‐sectional	10 (6/4)	10 (0/10)	53.5 (42–70)	27.1 (24–39)	P: BoP ≥ 25%, ≥ 2 teeth with CAL ≥ 4 mm, ≥ 2 teeth with PD ≥ 6 mm	H: no further specified	10 (50.0)	10 (50.0)	Partial‐mouth
Bostanci et al. (2018) [33]	İzmir, Turkey	Cross‐sectional	CP: 17 (7/10) AgP: 17 (6/11)	16 (5/11)	CP: 44.9 ± 8.2 AgP: 33.4 ± 5.4	34.1 ± 9.9	CP: > 4 non‐adjacent teeth with sites with CAL > 5 mm and PD > 6 mm, ABL > 50% in at least two quadrants AgP: CAL ≥ 5 mm and PD ≥ 6 mm on ≥ 8 teeth (at least 3 of these teeth other than central incisors or first molars), ABL > 30% on > 3 teeth other than first molars and incisors	G: BoP > 50%, no CAL > 2 mm, no radiographic ABL H: no sites with PD > 3 mm and CAL > 2 mm, BoP < 15%, and no detectable ABL	CP: 17 (34.0) AgP: 17 (34.0)	16 (32.0)	Full‐mouth
Mertens et al. (2018) [39]	Montpellier, France	Cross‐sectional	CP: 10 (6/4) AgP: 11 (6/5)	12 (4/8)	CP: 60.5 ± 9 AgP: 33.3 ± 9	26.3 ± 4	CP: Armitage [28] AgP: Armitage [28]	H: Armitage [28]	CP: 10 (30.3%) AgP: 11 (33.3%)	12 (36.4%)	Full‐mouth
Shin et al. (2019) [41]	YangPyeong, South Corea	Cross‐sectional	36 (24/12)	36 (24/12)	64.2 ± 9.1	64.2 ± 9.1	P: ABL ≥ 3 mm in ≥ 2 non‐ adjacent teeth (incipient P) or ABL ≥ 5 mm in ≥ 30% of teeth present (severe P)	H: absence of ABL (< 3 mm)	36 (50.0)	36 (50.0)	Full‐mouth (only panoramic radiographs assessment)
Tang et al. (2019) [42]	Beijing, China	Cross‐sectional	17 (8/9)	16 (5/11)	40.1 ± 11.6	33.1 ± 11.3	CP: CAL ≥ 1 mm, PD ≥ 4 mm, radiographic ABL, and > 30% of teeth involved	G: no sites with attachment loss, no sites with PD > 3 mm, BOP > 20%, and no radiographic ABL H: no sites with attachment loss, no sites with PD > 3 mm, BoP ≤ 20%, and no radiographic ABL	48 (57.8)	35 (42.2)	Full‐mouth
Antezack et al. (2020) [31]	Marseille, France	Cross‐sectional	67 (14/53)	74 (25/49)	50.2 ± 13.9	24.5 ± 3.3	P: Interdental CAL at 2 non‐adjacent teeth or buccal or oral CAL ≥ 3 mm with PD ≥ 3 mm at ≥ 2 teeth [29]	H: BoP < 10%, PD ≤ 3 mm and no clinical attachment loss [87]	67 (47.5)	74 (52.5)	Full‐mouth
Hartenbach et al. (2020) [38]	Rio de Janeiro, Brazil	Cross‐sectional	30 (16/14)	10 (3/7)	42.0 ± 2.6	29.9 ± 4.4	CP: > 10% of teeth with PD and/or CAL ≥ 5 mm and BoP	H: ≤ 10% of sites with BoP, no PD or CAL > 3 mm (PD or CAL = 4 mm without BoP allowed in up to 5% of the sites)	30 (75.0)	10 (25.0)	Full‐mouth
Grant et al. (2022) [37]	Birmingham and Newcastle upon Tyne, United Kingdom	Longitudinal (treatment)	MMP: 10 (5/5) AP: 10 (4/6)	10 (6/4)	MMP: 47 ± 6 AP: 49 ± 7	39 ± 9	MMP: interproximal CAL of 2–4 mm at > 8 teeth and PPD of 5–7 AP: interproximal CAL of > 5 mm at > 12 teeth and PPD of > 7 mm	G: no interproximal attachment loss, > 30% of sites with GI > 2, BoP > 30%, no sites with PPD > 4 mm H: no interproximal attachment loss, no PPD > 3 mm, < 10% sites with GI = 1 and no sites with GI = 2/3, < 10% sites with BoP	MMP: 10 (33.3) AP: 10 (33.3)	10 (33.3)	Full‐mouth
Casarin et al. (2023) [34]	São Paulo, Brazil	Longitudinal (treatment)	12 (2/10)	13 (2/11)	38.4 ± 4.2	37.0 ± 4.9	GAgP: ≥ 8 teeth with PD ≥ 5 mm (of which two must have PD ≥ 7 mm), BoP and CAL > 5 mm (at least 3 of these teeth other than central incisors or first molars), less than 35 years of age (at diagnosis)	H: PD < 4 mm, no radiographic ABL, no clinical attachment loss	12 (48.0)	13 (42.0)	Full‐mouth
Romano et al. (2023) [43]	Turin, Italy	Cross‐sectional	UP: 15 (5/10) TP: 15 (8/7)	15 (7/8)	UP: 48.5 ± 10.1 TP: 51.3 ± 8.5	43.3 ± 8.8	UP: stage III or stage IV generalized periodontitis [29] TP: reduced but stable periodontium (Chapple et al., 2018) after active periodontal treatment of stage III or stage IV generalized periodontitis	H: no loss of interdental CAL, no radiographic ABL, and FMBS < 10% [87]	UP: 15 (33.3) TP: 15 (33.3)	15 (33.3)	Full‐mouth

Abbreviations: ABL, alveolar bone loss; AgP, aggressive periodontitis; AP, advanced periodontitis; BoP, bleeding on probing; CAL, clinical attachment level; CP, chronic periodontitis; G, gingivitis; GAgP, generalized aggressive periodontitis; GI, gingival index; H, periodontally healthy individuals; MMP, mild to moderate periodontitis; NR, not reported; P, periodontitis patients; PD, pocket depth; TP, treated periodontitis; UP, untreated periodontitis.

Details about saliva collection and analytical protocols are summarized in Table 2. Information about pre‐sampling procedures (such as refraining from eating before saliva collection) were given in all the selected studies, except one. Nine studies collected unstimulated whole saliva; the remaining collected whole saliva after stimulation. Mass spectrometry was used coupled with liquid chromatography (LC–MS/MS) in eight articles. In the remaining five papers, salivary samples were analyzed by means of matrix‐assisted laser desorption ionization‐time of flight mass spectrometry (MALDI‐TOF MS).

**TABLE 2:** Methods of saliva collection and sample analysis across the included studies.

Authors	Pre‐sampling procedures		Saliva collection			Pre‐analytical procedures		Detection method
	Restrictions	Timing	Type of saliva	Volume	Time of sampling	Sample storage	Sample preparation	
Gonçalves et al. [36]	No tooth brushing Resting	2 h before 15 min before	Unstimulated saliva	NR	NR	−70°C	Yes	Two‐dimensional gel electrophoresis, MALDI‐TOF/TOF, LC–ESI–MS, nLC‐Q‐TOF
Salazar et al. [40]	NR	NR	Stimulated saliva	NR	NR	−80°C	Yes	LC–MS/MS
Chaiyarit et al. [35]	No eating and drinking	1 h before	Unstimulated saliva	3–5 mL	NR	−80°C	Yes	MALDI‐TOF MS
Belstrøm et al. [32]	No any dental treatment	NR	Stimulated saliva	NR	8–11 a.m.	−80°C	Yes	LC–MS/MS
Bostanci et al. [33]	No eating, drinking, tooth brushing, flossing, and mouth rinsing	2 h before	Unstimulated saliva	NR	8–10 a.m.	−80°C	Yes	LC–MS/MS
Mertens et al. [39]	No smoking No eating	4 h before 1 h before	Unstimulated saliva (water rinsing before collection)	3 mL	NR	−80°C	Yes	LC–MRM
Shin et al. [41]	No eating, drinking, and tooth brushing	1 h before	Unstimulated saliva	NR	NR	−80°C	Yes	LC–MS/MS, ELISA
Tang et al. [42]	No tooth brushing, flossing, mouth rinsing, and gum chewing	2 h before	Unstimulated saliva (water rinsing 10 min before collection)	NR	8–9 a.m.	−80°C	Yes	MALDI‐TOF MS, nano‐LC/ESI‐MS/MS
Antezack et al. [31]	No eating, drinking, and tooth brushing	1 h before	Unstimulated saliva	2 mL	NR	4°C	Yes	MALDI‐TOF MS
Hartenbach et al. [38]	No tooth brushing Resting	2 h before 15 min before	Stimulated saliva	NR	In the morning	−80°C	Yes	LC–MS
Casarin et al. [34]	NR	NR	Unstimulated saliva	NR	NR	−20°C	Yes	LC–MS/MS
Grant et al. [37]	No eating, drinking (except water), and tooth brushing	2 h before	Stimulated saliva	NR	NR	−80°C	Yes	LC–MS/MS
Romano et al. [43]	No eating, drinking, and tooth brushing	1 h before	Unstimulated saliva	5–6 mL	8–10 a.m.	−80°C	Yes	Two‐dimensional gel electrophoresis, MALDI‐TOF MS, Western‐Blot

Abbreviations: ELISA, enzyme‐linked immunosorbent essay; LC–ESI–MS, liquid chromatography—electrospray ionization—mass pectrometry; LC–MRM, liquid chromatography—multiple reaction monitoring; LC–MS, liquid chromatography—mass spectrometry; LC–MS/MS, liquid chromatography—tandem mass spectrometry; MALDI‐TOF MS, matrix‐assisted laser desorption ionization—time‐of‐flight mass spectrometry; MALDI‐TOF/TOF, matrix assisted laser desorption ionization—tandem time‐of‐flight; nano‐LC/ESI‐MS/MS, nano‐liquid chromatography—electrospray ionization—tandem mass spectrometry; nLC‐Q‐TOF, nano‐liquid chromatography—quadrupole—time‐of‐flight.

### 3.3 Salivary Proteomic Profiles in Periodontitis and Healthy Subjects

The results of the included studies are summarized in Table 3 and Table S2. The number of identified proteins ranged between 2 and 4161, and their quantities were presented as absolute values (expressed as Dalton and/or peaks), concentrations (μg/μL or pg/mL), or ratio (fold changes) between cases and controls. *p*‐values adjusted for false discovery rates (FDR) were reported in [32, 33, 38, 40, 42].

**TABLE 3:** Overview of the main findings of the included studies. Only proteins reported to be up‐ or down‐regulated by at least two studies are shown.

Authors	No of proteins identified	Reported unit of measure	Periodontitis (upregulated proteins)	Healthy controls (upregulated proteins)	Main findings
Gonçalves et al. [36]	27 (identified with nLC–MS/MS)	NR	Alpha‐amylase Serum albumin	Cystatin‐SN	Increased levels of some blood proteins and immunoglobulins and lower abundance of cystatin in periodontitis patients compared to the control group
Salazar et al. [40]	344	Fold changes between cases and controls	Adenylyl cyclase‐associated protein 1 Alpha‐2‐macroglobulin Catalase Complement C3 Fibrinogen alpha chain Gelsolin Lactotransferrin Matrix metalloproteinase‐9 Neutrophil collagenase Neutrophil defensin Peptidoglycan recognition protein 1 Plastin‐2 Profilin‐1 Protein S100‐P Rho GDP‐dissociation inhibitor 2	Lactoperoxidase	Proteomic analysis of whole saliva can differentiate between periodontitis and periodontal health. Potential biomarkers of periodontitis are related to the host's response to microbial challenge and inflammation
Chaiyarit et al. [35]	NR	Da (unidentified peaks)		5835.73 Da 9801.83 Da	MALDI‐TOF/TOF MS can be used to differentiate periodontitis from oral health and other oral diseases
Belstrøm et al. [32]	4161 (2090 human, 1946 bacteria)	NR	Alpha‐1B‐glycoprotein Apolipoprotein A Complement C3	Cell division control protein 42 homolog Keratin, type II cytoskeletal 1	Proteins related to inflammatory response and the complement system seem to be over‐expressed in periodontitis
Bostanci et al. [33]	360	Fold changes between cases and controls	Carbonic anhydrase 1 Profilin‐1 Protein S100‐A4 Protein S100‐A8	Alpha‐2‐macroglobulin‐like protein 1 Alpha‐amylase 2B Annexin A1 BPI fold‐containing family A member 2 Carbonic anhydrase 6 Cornulin Nystatin‐B Cystatin‐C Cystatin‐D Cystatin‐S Cystatin‐SA Nystatin‐SN Heat shock protein beta‐1 Leukocyte elastase inhibitor Prolactin‐inducible protein	A label‐free quantitative proteome analysis of saliva reveals 119 proteins with at least a 2‐fold significant difference between periodontal health and disease. External validation by targeted proteomics and machine learning modeling led to a panel of 5 proteins of high predictive value for periodontitis
Mertens et al. [39]	35	μg/μL	Hemopexin (HEMO) α‐fibrinogen (FIBA)	Apolipoprotein H (APOH) (only vs. CP) Plasminogen (PLMN)	LC–MRM could screen for periodontitis, with apolipoprotein H being a discriminant biomarker for aggressive periodontitis
Shin et al. [41]	744	pg/mL (ELISA)	Actin, aortic smooth muscle Alpha‐1B‐glycoprotein Apolipoprotein A‐I Complement C3 Fibrinogen alpha chain Fibrinogen beta chain Fibrinogen gamma chain Haptoglobin Heat shock 70 kDa protein 1A/1B, Neutrophil defensin 3 Prolactin‐inducible protein Protein S100‐A8 (verified with ELISA) Protein S100‐A9 (verified with ELISA) SPARC‐like protein 1	78 kDa glucose‐regulated protein Alpha‐2‐macroglobulin‐like protein 1 BPI fold‐containing family B member 1 Carbonic anhydrase 6 Cathepsin G Cystatin‐B Fructose‐bisphosphate aldolase Gelsolin Hemopexin Ig mu chain C region Immunoglobulin J chain (Fragment) Kallikrein‐1 Lactoperoxidase Neutrophil gelatinase‐associated lipocalin Peptidyl‐prolyl cis‐trans isomerase A Protein disulfide‐isomerase Protein S100‐A6 (Fragment)	Salivary S100A8 and S100A9 could be candidate biomarkers for periodontitis and be used for a point‐of‐care test
Tang et al. [42]	91	Da (peaks)	Haptoglobin		Mass spectrometry allows for the identification of potential biomarkers for chronic periodontitis in saliva, GCF, and serum
Antezack et al. [31]	217	Da (unidentified peaks)	3372 Da 3443 Da 3519 Da 3550 Da 6352 Da 6735 Da 12 692 Da 13 461 Da	2620 Da 7746 Da	A diagnostic test based on salivary protein profile diagnosed periodontitis with a sensitivity of 70.3% and a specificity of 77.8%
Hartenbach et al. [38]	473	Log2 transformed peak intensity	Cystatin‐SA Submaxillary gland androgen‐regulated protein 3B	Alpha‐2‐macroglobulin‐like protein Annexin A1 Apolipoprotein A‐I BPI fold‐containing family B member 1 Catalase Cathepsin G Cornulin Hemopexin Keratin, type I cytoskeletal 10 Keratin, type I cytoskeletal 13 Keratin, type I cytoskeletal 16 Keratin, type I cytoskeletal 9 Keratin, type II cytoskeletal 2 oral Keratin, type II cytoskeletal 4 Leukocyte elastase inhibitor Lipocalin‐1 (Tear lipocalin) Matrix metalloproteinase‐9	In chronic periodontitis salivary proteins with protective functions were significantly reduced, whereas few proteins related to inflammation and tissue destruction were increased
Grant et al. [37]	314	Ratio (periodontitis/health values)	Actin gamma 1 Carbonic anhydrase 1 MMP9 Plastin‐2 S100A8 S100A9	H vs. G: Keratin type II cytoskeletal 4 Pyruvate kinase	Quantitative mass‐spectrometry proteomics allowed for the identification of 95 proteins in both saliva and GCF; 15 candidate proteins were verified by ELISA and panels of 3–4 biomarkers were able to discriminate between periodontal health and disease states
Casarin et al. [34]	74	Normalized spectral counts pg/m	Alpha‐1‐antitrypsin Alpha‐amylase 1 Apolipoprotein A‐I Fibrinogen Heat shock protein beta‐1 Hemoglobin subunit alpha Keratin Lactoperoxidase Profilin‐1 Prolactin‐inducible protein Submaxillary gland androgen‐regulated protein 3B	Actin, cytoplasmic 1 Alpha‐2‐macroglobulin BPI fold‐containing family A member 2 Cystatin‐S Hemopexin IgGFc‐binding protein Immunoglobulin J chain Glucose‐6‐phosphate 1‐dehydrogenase Keratin, type I cytoskeletal 13 Keratin, type II cytoskeletal 4 Lactotransferrin (Fragment) Peptidyl‐prolyl cis‐trans isomerase	An altered salivary proteomic profile of children of periodontitis individuals as compared to descendants of periodontally healthy subjects was demonstrated. Annexin A1 was 7.1 times less produced in children of periodontitis individuals, being considered as a potential early biomarker for periodontitis
Romano et al. [43]	2	NR		Cystatin SN (CST1)	Salivary cystatin SN was markedly expressed in periodontally healthy individuals and absent in most periodontitis patients

Abbreviations: Da, Dalton; ELISA, enzyme‐linked immunosorbent essay; GCF, gingival crevicular fluid; LC–MRM, liquid chromatography multiple‐reaction monitoring; MALDI‐TOF/TOF MS, matrix‐assisted laser desorption and ionization (time‐of‐flight)^2^ mass spectrometry; nLC–MS/MS, nano‐liquid chromatography—tandem mass spectrometry; NR, not reported.

**FIGURE 2:**
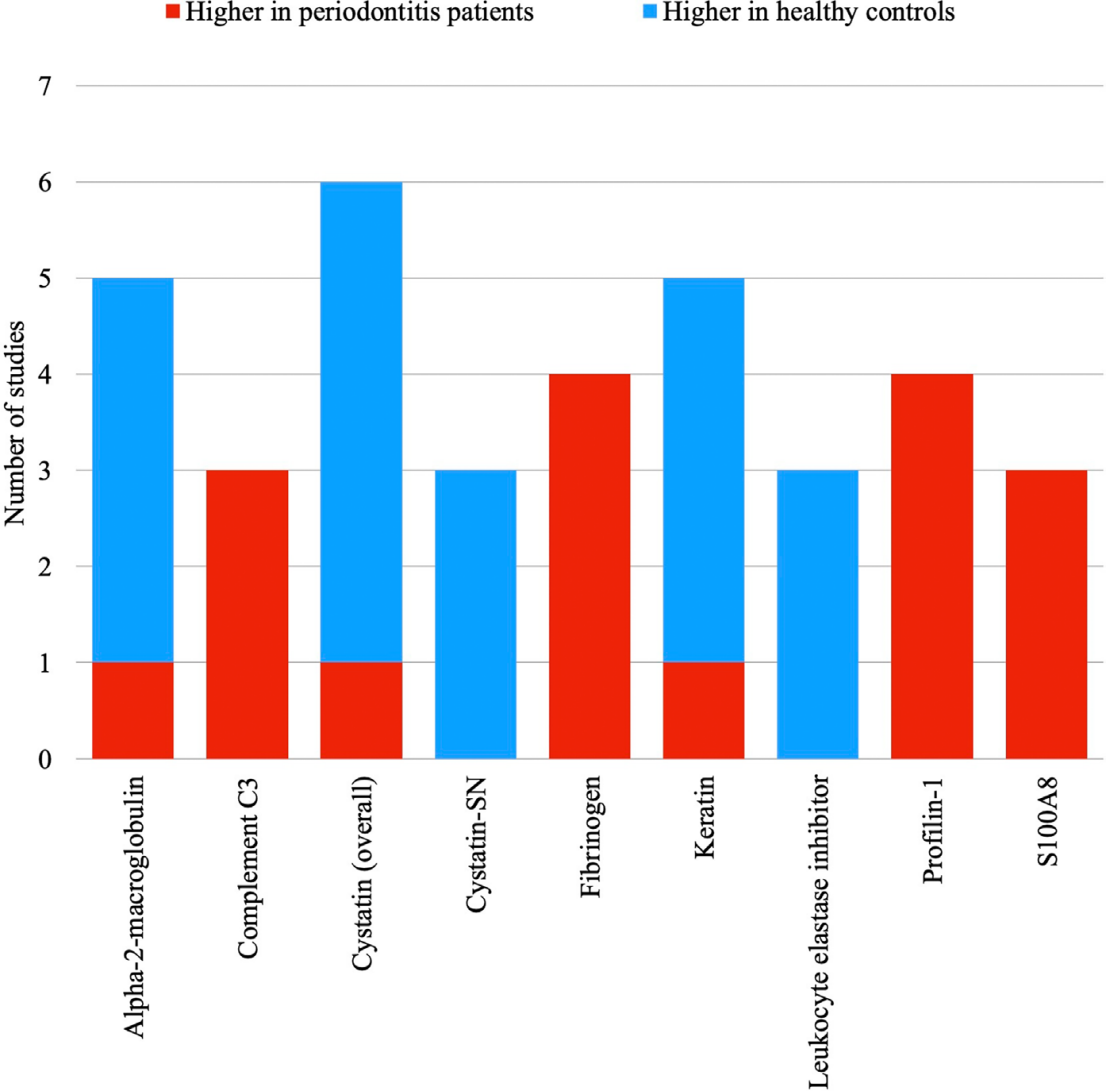
Consistency analysis for the differentially expressed proteins in saliva of patients with periodontitis compared to periodontally healthy controls. Only proteins consistently over‐expressed or under‐expressed in periodontitis patients in at least three studies were included.

Most of the included studies reported proteins differently expressed in cases and controls. Figure 2 illustrated the results of the consistency analysis. Conflicting results were reported for 17 out of the 52 proteins differently expressed in at least 2 studies. Complement C3, keratin (type I and type II), profilin‐1, S100A8, cystatin‐SN, alpha‐2‐macroglobulin, leukocyte elastase inhibitor, and fibrinogen were the proteins consistently over‐expressed or under‐expressed in periodontitis patients in at least three papers. Among them, complement C3, profilin‐1, S100A8, and fibrinogen were consistently increased in periodontitis cases. On the contrary, cystatin‐SN and leukocyte elastase inhibitor were consistently increased in periodontally healthy controls.

### 3.4 Diagnostic Accuracy

Analysis of diagnostic accuracy was reported in four of the included studies (Table S3). Antezack et al. [31] selected six unidentified protein peaks to build a diagnostic decision tree that identified periodontitis with sensitivity of 70.3% and specificity of 77.8%. In Bostanci et al. [33], machine‐learning modeling allowed to identify a group of five proteins (MMP‐9, Ras‐related protein‐1, Actin‐related protein 2/3 complex subunit 5, Clusterin, Deleted in Malignant Brain Tumors 1) with high predictive value for periodontitis [maximum area under the curve] (AUC) > 0.97. Grant et al. [37] selected a panel of 15 candidate proteins based upon differences observed with the untargeted analysis. The best performing group to distinguish between health (or gingivitis) and periodontitis resulted in an AUC of 0.970 and included alpha‐1‐acid glycoprotein, matrix metalloproteinase‐9, pyruvate kinase, S100A8, and age. They also tested the possibility to distinguish between mild‐to‐moderate and advanced periodontitis and obtained an AUC of 0.789. Tang et al. [42] analyzed the AUC of differentially expressed peptide peaks between chronic periodontitis and healthy groups, obtaining values ranging from 0.688 to 0.860.

When performing an additional literature search to assess whether other targeted works have reported on diagnostic utility measures for the identified biomarkers, only S100A8 was found in at least two different studies [19, 44]. Additional data on the diagnostic accuracy of S100A8 were retrieved from Grant et al. [37]. After meta‐analysis, the resulting AUC for this protein was 0.71 (95% CI: 0.66–0.75) with low heterogeneity (I2 = 2.4%, *p* = 0.359; Figure S1).

### 3.5 Pathway Enrichment Analysis

**FIGURE 3:**
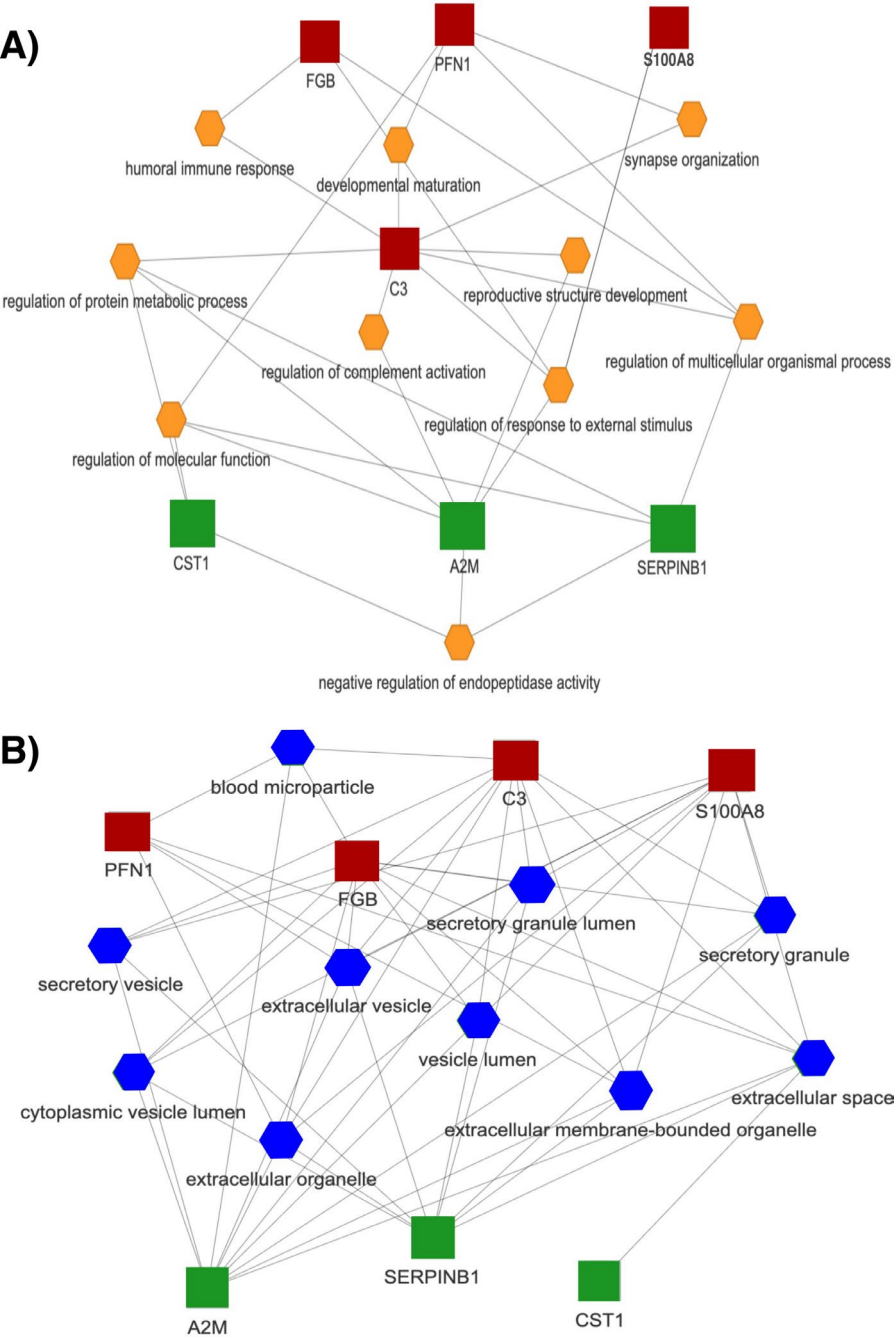
Functional enrichment analysis, using Flame database, of the common proteins identified in this systematic review with an integrated network of the top 10 enriched biological processes terms (A) and gene ontology cellular component (B) (P‐value < 0.05). Red squares = proteins up‐regulated in periodontitis; green squares = proteins up‐regulated in periodontally healthy controls.

Figure 3A depicts the top ten enriched networks of GO Biological Process associated with the most consistent proteins differentially expressed between cases and controls. Among the key biological processes shared by the selected molecules, the most common were: GO:0010951‐negative regulation of endopeptidase activity, GO:0032101‐regulation of response to external stimulus, GO:0006959‐humoral immune response, and GO:0030449‐regulation of complement activation. Similarly, the cellular components where the selected proteins are predominantly located are shown in Figure 3B, the main locations being the extracellular space or cell periphery, together with the secretory granules and vesicles.

### 3.6 Methodological Quality Assessment

Results of the methodological quality assessment are reported in Table 4. According to the total scores, two papers were classified as high quality, one as low quality and another one as very low quality. The remaining nine studies were classified as moderate quality. Most of the studies defined properly inclusion and exclusion criteria as well as case definitions of periodontitis and periodontal health. Only two studies reported the time period between the collection of salivary samples and the reference standard test (clinical and/or radiographic assessment of periodontal condition). The assessment of potential confounding factors (such as systemic diseases and smoking) was reported in most of the included studies. Importantly, results were validated in an independent cohort in only four papers. In particular, Bostanci et al. [33] carried out a selected‐reaction monitoring (SRM)‐targeted analysis in an independent cohort for validation of the results of the initial label‐free quantitative (LFQ) approach. In Casarin et al. [34], internal and external validations were performed for annexin A1 (ANXA1) using an enzyme‐linked immunosorbent assay (ELISA) test. Interestingly, the external validation was carried out in vitro using cell culture of gingival fibroblast. Grant et al. [37] selected a shortlisted potential biomarker to be verified by ELISA in a cohort consisting of the sample included in the initial analysis by quantitative mass spectrometry proteomics and another group of individuals recruited in a different center. In Shin et al. [41], MS analysis was followed by ELISA to verify the candidate protein markers (S100A8 and S100A9) among another cohort.

**TABLE 4:** Quality assessment of the included studies evaluated by a modified version of the QUADOMICS tool.

Item	Antezack et al. [31]	Belstrøm et al. [32]	Bostanci et al. [33]	Casarin et al. [34]	Chaiyarit et al. [35]	Gonçalves et al. [36]	Grant et al. [37]	Hartenbach et al. [38]	Mertens et al. [39]	Romano et al. [43]	Salazar et al. [40]	Shin et al. [41]	Tang et al. [42]
1. Was the research question or objective in this paper clearly stated and appropriate?	Yes	Yes	Yes	Yes	Yes	Yes	Yes	Yes	Yes	Yes	Yes	Yes	Yes
2. Was the study population clearly specified and defined?	Yes	Yes	Yes	Yes	No	Yes	Yes	Yes	Yes	Yes	Yes	No	Yes
3. Were the procedures and timing of biological sample collection with respect to clinical factors described with enough detail?	0.75	0.50	1	0.25	0.25	0.75	0.75	0.75	0.25	1	0	0.75	0.75
4. Did the authors include a sample size justification?	No	No	NR	No	No	No	No	No	No	No	Yes	Yes	No
5. Were controls selected or recruited from the same or similar population that gave rise to the cases (including the same timeframe)?	Yes	Yes	Yes	Yes	NR	Yes	Yes	Yes	Yes	Yes	Yes	Yes	Yes
6. Were the definitions, inclusion and exclusion criteria, used to identify or select cases and controls valid, reliable, and implemented consistently across all study participants?	Yes	Yes	Yes	Yes	No	Yes	Yes	Yes	No	Yes	Yes	Yes	Yes
7. Were the cases clearly defined and differentiated from controls?	Yes	Yes	Yes	Yes	No	Yes	Yes	Yes	Yes	Yes	Yes	Yes	Yes
8. Were handling of specimens and pre‐analytical procedures reported in sufficient detail and similar for the whole sample?	1	1	1	0.50	0.50	1	1	1	0.25	1	0.5	1	1
9. Is the time period between the reference standard and the index test short enough to reasonably guarantee that the target condition did not change between the two tests?	Unclear	Unclear	Unclear	Unclear	Unclear	Unclear	Yes	Unclear	Unclear	Yes	Unclear	Unclear	Unclear
10. Is the reference standard likely to correctly classify the target condition?	Yes	Yes	Yes	Yes	Unclear	Yes	Yes	Yes	Yes	Yes	Yes	No	Yes
11. Did the whole sample or a random selection of the sample receive verification using a reference standard of diagnosis?	Yes	Yes	Yes	Yes	Yes	Yes	Yes	Yes	Yes	Yes	Yes	Yes	Yes
12. Were key potential confounding variables measured and adjusted statistically in the analyses? If matching was used, did the investigators account for matching during study analysis?	1	1	1	1	0.50	1	1	1	0	1	1	0.50	1
13. Were uninterpretable/intermediate test results reported?	No	No	No	No	No	No	No	No	No	No	No	No	No
14. Were untargeted results validated using more sensitive methods (i.e., ELISA)?	No	No	Yes	Yes	No	No	Yes	No	No	No	No	Yes	No
15. Were the findings validated externally using an independent cohort?	No	No	Yes	No	No	No	Yes	No	No	No	No	Yes	No
Total score	9.75	9.50	12	10.75	3.25	9.75	12.75	9.75	6.50	11	9.50	11.25	9.75
Quality evaluation	Mod.	Mod.	High.	Mod.	Very Low	Mod.	High.	Mod.	Low	Mod.	Mod.	Mod.	Mod.

Abbreviations: Mod., moderate; NR, not reported.

## 4 Discussion

This systematic review, which includes 13 original articles, demonstrates that distinctive molecular signatures identified through proteomic methods have the potential to differentiate between patients with periodontitis and controls with periodontal health or gingivitis. The data provide aggregated evidence of novel candidate biomarkers that could be exploited for future diagnostic tools to screen and assess periodontitis. Below, a biological background and an interpretation of the most significantly, differentially expressed molecules among the clinical groups are provided, along with a discussion of the methodologies used.

### 4.1 Outcomes for Specific Proteins

#### 4.1.1 Complement C3

Complement C3 is a protein of the innate immune system which plays a central role in the activation of the complement system by the classical, lectin and alternative pathways. Complement dysregulation can be caused by genetic or microbial factors and it is regarded as a contributing factor in the pathogenesis of several disorders, including periodontitis. In a pre‐clinical study, C3‐deficient mice did not develop gingival inflammation and periodontal bone loss [45]. In humans, it was reported the presence of complement metabolites in the GCF during periodontal tissues inflammation [46, 47], whereas periodontitis treatment was associated with reduced complement C3 activation [48]. Recently, Cp40 (an analog to C3‐inhibiting compstatin) was investigated for the treatment of gingival inflammation and periodontitis in both animal models and humans [49, 50, 51]. Cp40 is able to interrupt the complement cascade by blocking the binding of C3 to its convertase, making this molecule a potential adjunctive treatment option for periodontitis [52]. In the present systematic review, complement C3 was constantly over‐expressed in periodontitis patients, supporting the central role of this protein in the alterations of the immune system related to the onset and progression of periodontitis.

#### 4.1.2 Profilin‐1

Four out of the 13 studies included in our systematic review reported higher profilin‐1 levels in periodontitis patients as compared to periodontally healthy controls [33, 34, 37, 40]. Profilin‐1 is an actin monomer‐binding protein encoded by the PFN1, which is involved in human mesenchymal stem cells migration and proliferation by increasing the level and organization of cytoskeletal filamentous‐actin as a response to increased levels of pro‐inflammatory mediators such as prostaglandin E_2_ [53]. Even if the role of profilin‐1 within the periodontal tissues inflammatory processes has not been fully clarified, its higher expression increases endothelial cell permeability and ICAM‐1 expression [54]. In virtue of these characteristics, profilin‐1 has been also hypothesized as biomarker for atherogenesis, diabetes, and myocardial infarction [55].

#### 4.1.3 S100A8

S100A8 belongs to the family of S100 proteins, low molecular‐weight proteins characterized by two calcium‐binding sites. S100 proteins take part in a number of intracellular and extracellular processes, such as nervous system development, cell growth and differentiation, cell–cell communication, protein phosphorylation, calcium homeostasis, and the inflammatory response [56]. S100A8 (also known as calgranulin A) is mainly expressed on neutrophils, monocytes, and macrophages, it is involved in regulating inflammatory responses and has potent anti‐oxidant activity [57]. S100A8 forms a heterocomplex with S100A9 (calgranulin B) called calprotectin, but these calcium‐binding proteins have actions that are both dependent on or independent of heterocomplex formation. The S100A8/A9 complex inhibits the activity of matrix metalloproteinases [58] and has been used as a marker for rheumatoid arthritis [59], colorectal cancer, inflammatory bowel disease [60], and periodontitis [61]. Recently, three targeted studies demonstrated significantly higher salivary levels of S100A8 in periodontitis patients as compared to healthy controls [19, 44, 62]. In this systematic review, three out of the 13 included studies reported higher S100A8 levels in periodontitis patients as compared to periodontally healthy controls [33, 37, 41].

#### 4.1.4 Fibrinogen

Fibrinogen (coagulation factor I) is a 340 kDa glycoprotein complex that is converted enzymatically by thrombin to fibrin, the main component of blood clot. Fibrinogen is long known to act as an acute‐phase protein and participates in systemic and localized reactions in response to infections and inflammatory process [63, 64]. Indeed, its levels can be elevated up to 10‐fold during inflammatory conditions as compared to basal levels [65], and it was also demonstrated that over‐production of fibrinogen can increase the level of inflammatory cytokines and promote bacterial colonization [66, 67]. Periodontitis patients exhibit higher blood levels of C‐reactive protein and fibrinogen [68, 69], and these systemic alterations were linked with the risk of myocardial infarction and other cardiovascular diseases [70, 71]. It was also demonstrated that patients with genetically determined higher levels of plasmatic fibrinogen were at higher risk of having periodontitis [72]. The present data demonstrated that higher levels of fibrinogen (alpha, beta, and gamma chains) were consistently found in salivary samples of periodontitis patients than in controls [34, 39, 40, 41]. This is not surprising considering that inflamed periodontal tissues are sites of continuous local activation of the coagulation cascade. However, despite the interaction between pro‐inflammatory cytokines and fibrinogen levels, a potential role of fibrinogen in the pathogenesis of periodontitis has not been clarified. It is interesting to note that, looking at the consistency analysis, fibrinogen resulted to be the most frequently over‐expressed protein in salivary samples of periodontitis patients, making this molecule an interesting potential biomarker of periodontal disease. However, it should be considered that fibrinogen over‐expression could also be the result of gingival inflammation without involvement of deep periodontal tissues and subsequent loss of attachment.

#### 4.1.5 Cystatin‐SN

The cystatin isoforms A, B, C, D, SN, and SA were detected in saliva by high‐resolution mass spectrometry [73]. The main function of salivary cystatins is to inhibit cysteine proteases, such as bacterial proteases and lysosomal cathepsins and matrix metalloproteases, that are regarded as major contributors to periodontal tissue degradation [74, 75]. Since this proteolytic activity is mainly regulated by inhibitory proteins produced by the host, salivary cystatins have been investigated as potential biomarkers of active periodontitis. Beside the role as major inhibition of cysteine proteases, cystatin SN participates in important signal transduction pathways, such as WNT, GSK‐3, and interleukin‐6 signaling pathways, involved in inflammatory processes, immunity, and cell cycle regulation [76]. In a cross‐sectional study, the concentration of cystatin SN (encoded by the CST1 gene on chromosome 20p11.2) was found to be depleted in periodontitis patients as compared to healthy controls, but still present in sufficient quantities to inhibit cathepsins H and L [77]. According to the consistency analysis, cystatin SN was reported to be significantly elevated in healthy controls as compared to periodontitis patients in 3 out of the 13 studies included in our systematic review. In particular, this difference was reported between healthy controls and both aggressive [33] and chronic periodontitis patients [36]. The reduction of salivary cystatin SN in periodontitis patients could be associated with greater proteolytic damage to the periodontal tissues, with a subsequent progression of periodontal disease. In agreement with this hypothesis, Romano et al. [43] reported that cystatin SN expression in a group of treated periodontitis patients (reduced but stable periodontium) was re‐established to levels comparable to that of periodontally healthy subjects, suggesting that this protein could be used not only as a biomarker to support the diagnosis of periodontitis but also to monitor treatment response and risk for disease progression [78].

#### 4.1.6 Leukocyte Elastase Inhibitor

Leukocyte elastase inhibitor (LEI) is a member of the serine protease inhibitors superfamily, typically expressed in granulocytes and macrophages. This intracellular protein protects cells from proteases released into the cytoplasm during stress or infection. LEI is able to control cell survival showing both pro‐ and anti‐apoptotic properties [79]. It is also involved in the regulation of the innate immune response, in the resolution of chronic inflammation, in cell migration, and in wound healing. Three out of the included studies in the present systematic review reported that salivary levels of LEI was increased in healthy subjects as compared to periodontitis patients. Even if we have no information about the potential role of this protein in the etiopathogenesis of periodontitis, it is possible to speculate that its depletion in periodontitis patients is associated with an impairment in immune response against the periodontal pathogens as well as in wound healing, as confirmed by the pathway enrichment analysis.

### 4.2 Methodologic Considerations

From a methodological standpoint, the present review shows that salivary proteomics is a suitable method for identifying biomarkers that are correlated with the health status of periodontal tissues. Specifically, the untargeted proteomic approach is shown to offer several advantages over targeted techniques. First, it enables the identification of novel biomarkers previously unassociated with periodontal diseases. Additionally, this technique is able to capture a wide spectrum of proteins even those present at low abundance or post‐translationally modified, providing a deeper understanding of diseases. Advanced mass spectrometry techniques used in untargeted proteomics offer deep proteome coverage, enabling the detection of thousands of proteins in a single analysis. This high throughput capability is crucial for discovering biomarkers in complex biological samples such as oral tissues and fluids [80]. In addition, contemporary untargeted proteomics offers quantitative data, allowing for the comparison of protein expression between healthy and diseased states, which is crucial for identifying differentially expressed biomarkers [81]. Notably, to obtain reliable quantitative outcomes it is still necessary to design an analytical method which incorporates calibration analytes and multiple quality control steps in the study. A calibration curve is established by the analysis of the calibration analytes at known concentration, and this curve can be used to determine the concentrations of target compounds. Moreover, quality control steps are conducted at regular intervals to identify potential instrument biases and ensure that the quantitative data remain accurate [82].

This review showed that LC–MS/MS was the method of choice for the proteomic analysis of oral biofluids. LC–MS/MS is characterized by an elevated sensitivity and specificity together with the capability to handle complex protein digests. Furthermore, this technique can provide essential information such as proteins' post‐translational modifications and structural characteristics which can enhance a deeper understanding of the molecular pathways underlying periodontal diseases. An alternative mass spectrometry approach that proved its feasibility for the discovery of periodontal diseases biomarkers is MALDI‐TOF, which is well known for its speed and high‐throughput analysis of biomolecules. However, in order to get sufficient resolution and protein identification, it is typically necessary to preceding protein separation by two‐dimensional gel electrophoresis. This extra step introduces laborious pre‐analytical procedures and time consumption. Overall, LC–MS/MS represents the optimal technique for analyzing oral biofluids, offering comprehensive and high‐resolution proteomic data without requiring labor‐intensive pre‐analytical steps [83, 84]. LC–MS/MS revolutionized both research and clinical laboratories; however, some limitations are present, especially the lack of standardized instrument configuration since most of the LC–MS/MS methods used have been designed “intra‐laboratory.” From a strictly analytical standpoint, the main challenges to overcome relies on the possible compound misidentification due to Isobaric Interference. Furthermore, low‐abundance molecules might sometimes be hardly detectable without pre‐concentration or enrichment steps [24].

Some hurdles need to be solved to bring LC–MS/MS into the daily clinical practice. The purchase and maintenance of an LC–MS/MS is still expensive. Furthermore, the operation of the instrument and the complex data analysis can be performed only by highly skilled personnel. Another critical aspect is the translatability of LC–MS/MS into a “point of care” technique. Despite its potential, several challenges must be addressed for this transition to be possible. Specifically, advances in miniaturization, cost reduction, and the development of user‐friendly interfaces are essential to make LC–MS/MS a viable option for rapid, on‐site applications.

### 4.3 Limitations and Future Research Perspectives

The data resulting from this systematic review should be interpreted with some caveats. Indeed, most of the included studies were classified as moderate quality, only two as high quality, and the remaining as low or very low quality. Moreover, several definitions of cases and controls were adopted among the selected studies, resulting in a broad range of disease severity, inflammation degree, and number of involved teeth/sites. Another critical issue was the heterogeneity of the reported results (absolute values expressed as Dalton and/or peaks, concentrations, or ratio), preventing the possibility of summarizing data in a quantitative meta‐analysis. It is also important to underline that only four studies [33, 34, 37, 41] carried out external validation of untargeted findings on a separate cohort and that in one case validation was the result on an in vitro assay [34]. Validation, particularly external validation, is a critical aspect of research focused on developing diagnostic and screening tests. Without rigorous external validation, there is a risk that findings may not generalize beyond the original study population, potentially limiting the clinical utility and reliability of the biomarkers identified. Also, in light of the high‐dimensional nature of proteomic data, particularly with methods such as LC–MS, adjusting p‐values for FDR is crucial to minimize the risk of identifying false positives. Many of the included studies did not explicitly report FDR‐adjusted values, which could affect the reliability of the identified biomarkers. As such, the absence of FDR corrections in some studies must be considered a limitation, underscoring the importance of adopting FDR adjustments in future proteomic research to enhance the robustness of biomarker identification.

Another aspect to consider was the method for saliva collection. Nine out of the 13 included studies reported that salivary collection was unstimulated just because no mechanical devices or chemicals were given to the participants to stimulate salivary production. This unstimulated protocol allows to standardize the salivary flow rate minimalizing its impact on salivary protein concentration. However, none of the described procedure mentioned the required absence of several other sensory stimuli (such as room light, smell, noise, and temperature) that may alter the quantity and quantity of salivary production. Even the contraction of facial muscles may be considered a relevant factor and donors must be instructed to “drool” saliva into the sterile tube avoiding any movement during the procedure [85]. For these reasons, none of the selected study carried out salivary collection in a truly unstimulated modality.

A relevant point also relates to the confounding effect introduced by gingivitis in the proteomic profile. For instance, despite the discriminant molecules were also significant for periodontitis alone, Grant et al. [37] reported that profilin‐1 and S100A8 were elevated in gingivitis compared to periodontally healthy subjects. This is not surprising, as gingivitis and periodontitis are considered as a continuum [86]. However, an ideal biomarker for periodontitis should specifically indicate active periodontal tissue destruction, as opposed to marginal gingival inflammation. To mitigate this potential overlap, we grouped gingivitis with controls, as outlined in the PECO. While this approach may have clustered some inflammatory markers into the healthy group, it enhances the classifier's utility in clinical practice, where it is rare to encounter patients with less than 10% FMBS. Since only three out of the studies included in this review specifically separated gingivitis from health, and none offered a direct comparison between periodontitis and gingivitis, it remains unclear which proteins can be specific for gingival inflammation without attachment loss.

Finally, the majority of the reviewed articles focused on basic discovery research and rarely address the translation into clinical practice. To bridge the gap between research and clinical application, the proposed techniques should provide evidence of analytical sensitivity, analytical specificity, precision (repeatability and reproducibility), accuracy, limits of detection, quantitation, and measuring range. Future studies need to address all these criteria to meet the standards set by regulatory agencies for in vitro diagnostic (https://health.ec.europa.eu/medical‐devices‐sector/new‐regulations_en).

## 5 Conclusions

### 5.1 Implications for Clinical Practice

According to the results of the present systematic review including untargeted proteomics studies, an array of salivary proteins over‐ or under‐expressed in periodontitis patients as compared to healthy controls was identified. These proteins, involved in the regulation of the inflammatory response and tuning of the immune system, were consistently reported in at least 3 studies out of the 13 selected. The possibility of using one of these proteins or a combination to provide a salivary screening test for periodontitis and to support early diagnosis may support future clinically translatable approaches.

### 5.2 Implications for Research

Among the included studies, methods and reporting were heterogeneous, highlighting the need to develop and implement standardized protocols in future research. Notably, very few of the identified potential biomarkers were evaluated for basic diagnostic accuracy measures, underscoring the need for future targeted approaches. These findings should be validated and confirmed in larger comparative and longitudinal studies to provide more consistent data on the diagnostic utility of the identified markers, as well as to assess the impact of periodontal therapy on protein expression. Finally, potential biomarkers should be tested for their ability to discriminate between periodontitis and gingivitis.

## Author Contributions

M.C., G.B., F.R., and M.A. made substantial contributions to conception of the study. M.C., G.B., and G.N.B. contributed to the study design. M.C., G.B., and G.I. searched and collected the data. M.C., G.B., F.F., G.N.B., and F.R. performed data analysis and interpretation. M.C., G.B., F.F., J.Z., and F.R. prepared the first draft of the manuscript. All authors have read, revised critically, and approved the final manuscript.

## Ethics Statement

The authors have nothing to report.

## Consent

The authors have nothing to report.

## Conflicts of Interest

The authors declare no conflicts of interest.

## Supporting information


Figure S1.

Table S1.

Table S2.

Table S3.

## Data Availability

The data that support the findings of this study are listed in the main manuscript and Supporting Information.

## Appendix 1

An additional literature search to evaluate the diagnostic ability of the identified markers (complement C3, cystatin SN, fibrinogen, leukocyte elastase inhibitor, profilin‐1, S100A8) was performed on Medline via PubMed, using the following combinations of controlled terms (MeSH and EMTREE) and keywords:

1. “periodont*” AND “complement C3” AND (“diagnostic accuracy” OR “sensitivity” OR “specificity” OR “area under the curve” OR “AUC” OR “receiver operating characteristic” OR “ROC”). This search resulted in three titles, with no one reporting data on the diagnostic accuracy of salivary complement C3.
2. “periodont*” AND “cystatin SN” AND (“diagnostic accuracy” OR “sensitivity” OR “specificity” OR “area under the curve” OR “AUC” OR “receiver operating characteristic” OR “ROC”). No titles were obtained.
3. “periodont*” AND “fibrinogen” AND (“diagnostic accuracy” OR “sensitivity” OR “specificity” OR “area under the curve” OR “AUC” OR “receiver operating characteristic” OR “ROC”). This search resulted in 51 titles, four were selected for full‐text reading but no relevant data on diagnostic accuracy were reported.
4. “periodont*” AND (“leukocyte elastase inhibitor” OR “LEI”) AND (“diagnostic accuracy” OR “sensitivity” OR “specificity” OR “area under the curve” OR “AUC” OR “receiver operating characteristic” OR “ROC”). Four titles were obtained, yet excluded after screening.
5. “periodont*” AND “profilin‐1” AND (“diagnostic accuracy” OR “sensitivity” OR “specificity” OR “area under the curve” OR “AUC” OR “receiver operating characteristic” OR “ROC”). This search resulted in 2 titles, with no one reporting data on the diagnostic accuracy of profilin‐1 in saliva.
6. “periodont*” AND (“S100A8” OR “calgranulin A” OR “myeloid‐related protein 8” OR “MRP8”) AND (“diagnostic accuracy” OR “sensitivity” OR “specificity” OR “area under the curve” OR “AUC” OR “receiver operating characteristic” OR “ROC”). Seven titles were screened and four studies were selected for full‐text reading. Finally, two papers were included to evaluate the diagnostic ability of S100A8 [19, 44]. Another study [37] has been already included in the systematic review.
