# Supplementary figures and images for: Salivary Proteomics for Detecting Novel Biomarkers of Periodontitis: A Systematic Review
Source: J Periodontal Res. 2024 Dec 2;60(7):633–55. doi: 10.1111/jre.13357 (PMC12371805; doi:10.1111/jre.13357)

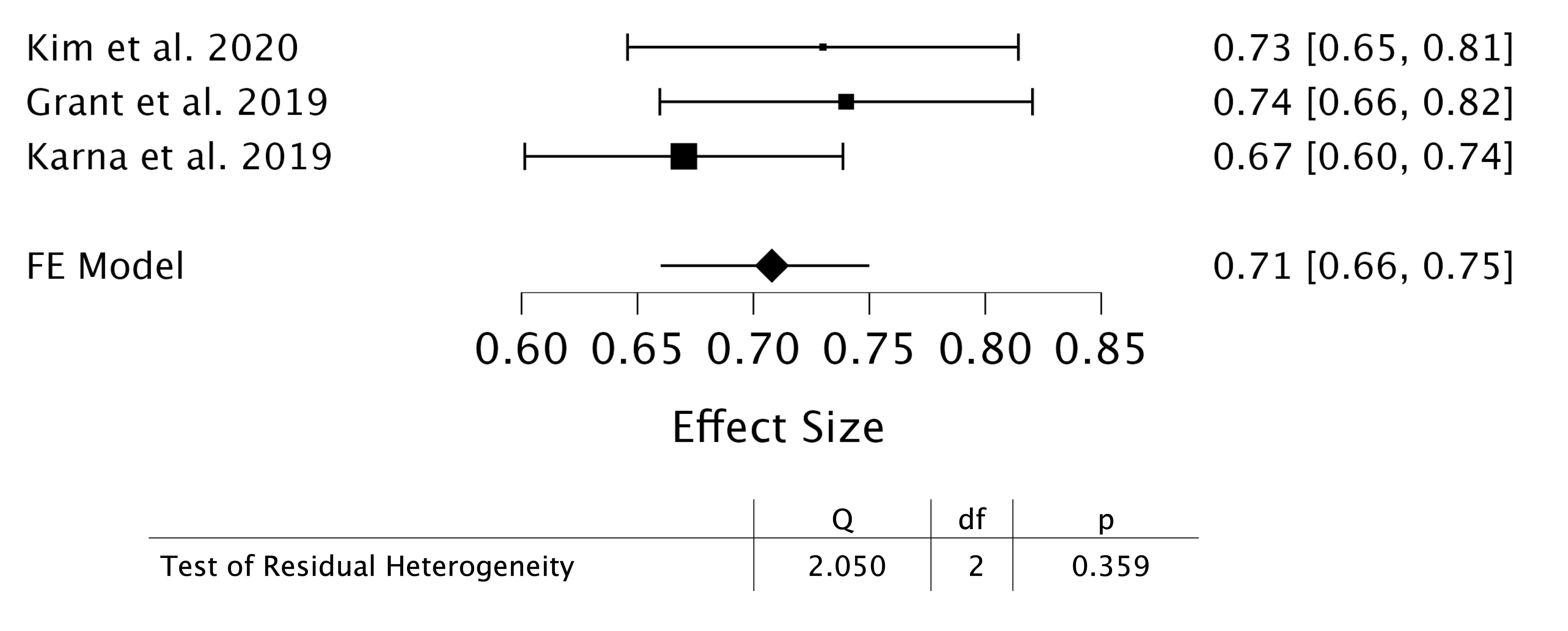

Supplement: Supplementary file 1 — Figure S1. [file JRE-60-633-s001.tiff]
