# Supplementary material for: Salivary Proteomics for Detecting Novel Biomarkers of Periodontitis: A Systematic Review
Source: J Periodontal Res. 2024 Dec 2;60(7):633–55. doi: 10.1111/jre.13357 (PMC12371805; doi:10.1111/jre.13357)
Supplement: Supplementary file 2 — Table S1. [file JRE-60-633-s003.docx]

**Supplementary Figure S1.** The resulting AUC for this protein with low heterogeneity.

**Supplementary Table S1.** Studies excluded after full text reading, with reason for exclusion.

| Reference | Reason For Exclusion |
| --- | --- |
| Wilmarth et al. (2004) | 1 |
| Wu et al. (2009) | 1 |
| Haigh et al. (2010) | 3 |
| Kim et al. (2010) | 1 |
| Zhang et al. (2012) | 1 |
| Recker et al. (2015) | 6 |
| Trindade et al. (2015) | 1 |
| Wu et al. (2016) | 1 |
| Haririan et al (2018) | 2 |
| Helmerhorst et al. (2018) | 2 |
| Grant et al. (2022) | 2 |
| Beyer-Hans et al. (2020) | 1 |
| Huang et al. (2020) | 4 |
| Kurgan et al. (2022) | 2 |
| Orti et al. (2018) | 5 |
| Pacheco et al. (2022) | 1 |
| Şengül et al. (2022) | 2 |
| Wang et al. (2022) | 4 |
| Yilmaz et al. (2022) | 2 |
| Yuan at al. (2022) | 3 |
| Chaiben et al. (2023) | 6 |
| Önder et al. (2023) | 2 |
| Sales-Peres et al. (2023) | 6 |
| Téllez Corral et al. (2023) | 2 |

**Reason for exclusion (*Legend*)**

- 1. Less than 10 patients per group
  2. Targeted analysis
  3. No control group
  4. Not focused on salivary proteins
  5. Duplicate
  6. Only systemically diseased patients
