# Supplementary material for: Salivary Proteomics for Detecting Novel Biomarkers of Periodontitis: A Systematic Review
Source: J Periodontal Res. 2024 Dec 2;60(7):633–55. doi: 10.1111/jre.13357 (PMC12371805; doi:10.1111/jre.13357)
Supplement: Supplementary file 3 — Table S2. [file JRE-60-633-s002.docx]

**Supplementary table S2** Overview of the main findings of the included studies

| Authors (year) | N° of proteins identified | Unite of measure | Difference in specific protein | | |
| --- | --- | --- | --- | --- | --- |
|  |  |  | Periodontitis (upregulated proteins) | Gingivitis (upregulated protein) | Healthy controls (upregulated proteins) |
| Gonçalves et al. (2010) | 27 (identified with nLC-MS/MS) | NR | Serum albumin  Alpha-amylase |  | Cystatin-SN |
| Salazar et al. (2013) | 344 | Fold changes between cases and controls | Protein S100-P  Ceruloplasmin  Alpha-2-HS-glycoprotein  Complement C3  Alpha-2-macroglobulin  Fibrinogen alpha chain  Plastin-2  Calreticulin  Lactotransferrin  Profilin-1  Gelsolin  Peptidoglycan recognition protein 1  Neutrophil defensin  Neutrophil collagenase  Matrix metalloproteinase-9  Rho GDP-dissociation inhibitor 2  Catalase  Adenylyl cyclase-associated protein 1  Leukotriene A-4 hydrolase |  | **Lactoperoxidase** |
| Chaiyarit et al. (2015) | NR | Da (peaks) |  |  | 5,835.73 Da  9,801.83 Da |
| Belstrøm et al. (2016) | 4,161 (2,090 human, 1,946 bacteria) | NR | Caspase-14  N-acetylmuramoyl-L-alanine amidase  Apolipoprotein B-100  Serum amyloid A-4 protein  Thyroxine-binding globulin  Complement C4-B  Retinol-binding protein 4  Ig heavy chain V-III region KOL  Apolipoprotein A  Plasma protease C1 inhibitor  Complement C4-A  Inter-alpha-trypsin inhibitor heavy chain H2  Complement factor H  Alpha-1B-glycoprotein  Beta-2-glycoprotein 1  Complement C3  Complement C1q subcomponent subunit A  Complement component C8 gamma chain  Apolipoprotein E |  | Ribosyldihydronicotinamide dehydrogenase [quinone]  Epiplakin  Protein-tyrosine kinase 6  Proteasome activator complex subunit 3  Keratin, type II cytoskeletal 1  Phosphoribosyl pyrophosphate synthase-associated protein 1  Epsin-3  Arachidonate 12-lipoxygenase, 12R-type  Nucleophosmin  Calcium/calmodulin-dependent protein kinase type II  Eukaryotic translation initiation factor 2A  Polypyrimidine tract-binding protein 38  Transmembrane protein 40  Ras GTPase-activating protein-binding protein 1  Very-long-chain 3-oxoacyl-CoA reductase0  Cell division control protein 42 homolog  F-actin-capping protein subunit alpha-2  Tyrosine-protein kinase CSK |
| Belstrøm et al. (2016) |  |  | Complement C5  Fibronectin |  | Carcinoembryonic antigen-related cell adhesion molecule 7  Serine/arginine-rich splicing factor 3  E3 ubiquitin-protein ligase HUWE1  Eukaryotic translation initiation factor 17  Retinol-binding protein 1  Eukaryotic translation initiation factor 3 subunit D  3'(2'),5'-bisphosphate nucleotidase 1  Protein transport protein Sec31A  Stromal interaction molecule 1  Cytosolic acyl coenzyme A thioester hydrolase  RNA-binding protein 47 |
| Bostanci et al. (2018) | 360 | Fold changes between cases and controls (?) | Highest mean in CP:  Band 3 anion transport protein  Ribonuclease R [Porphyromonas gingivalis ATCC 33277]  Carbonic anhydrase 1  Ras GTPase-activating-like protein IQGAP1  Synaptotagmin-5  Proteasome activator complex subunit 2  Metallo-beta-lactamase [Treponema denticola ATCC 35405]  Beta-casein, Bos taurus (Bovine)  Highest mean in AgP:  cDNA FLJ50152, highly similar to Synaptotagmin-5  Putative high mobility group protein B1-like 1  Ras GTPase-activating-like protein IQGAP1  Hemoglobin subunit alpha  Glutaredoxin-1  Carbonic anhydrase 1  Protein S100-A4  Hypoxanthine-guanine phosphoribosyltransferase  Non-histone chromosomal protein HMG-17  Ras-related protein Rap-1A  Serpin B10  Band 3 anion transport protein  Proteasome activator complex subunit 2  Fc-gamma receptor IIIb (CD 16)  Coactosin-like protein |  | Highest mean in H as compared to CP:  Isocitrate dehydrogenase [NADP] cytoplasmic  Isoform 1 of Serpin  Isoform 1 of Histone deacetylase  Calmodulin-like protein 5  Isoform 1 of Phospholipid transfer protein  Titin, isoform CRA_a  Interleukin-36 alpha  Isoform 1 of Alpha-1-antichymotrypsin  Annexin A1  Cellular retinoic acid-binding protein 2  Antileukoproteinase  Putative uncharacterized protein BUD2 OS=Candida albicans  Isoform 2A of Desmocollin-2  Isoform 1 of Carboxylesterase 2  Basic salivary proline-rich protein 2  Isoform 1 of Extracellular matrix protein 1  Monocyte differentiation antigen CD14  Alpha-amylase 2B  Immunoglobulin heavy variable 3-7  Elafin  Cornifin-B  Cystatin-B  Alpha-2-macroglobulin-like protein 1  Isoform 2 of Cell division control protein 42 homolog  Isoform 1 of Alpha-1-antitrypsin  Protein FAM3D  Tubulin beta-4B chain  Histatin-1  Leukocyte elastase inhibitor  Isoform 1 of WAP four-disulfide core domain protein 2  Thioredoxin |
| Bostanci et al. (2018) |  |  | Actin, alpha cardiac muscle 1  Isoform Short of Glucose-6-phosphate 1-dehydrogenase  Profilin-1  Actin-related protein 2/3 complex subunit 1B  Isoform 1 of Adenylyl cyclase-associated protein 1  Myeloid cell nuclear differentiation antigen  Isoform GN-1L of Glycogenin-1  Isoform 1 of Neutrophil gelatinase-associated lipocalin  Vasodilator-stimulated phosphoprotein  Adenylyl cyclase-associated protein  Protein S100-A8  Thymosin beta-4-like protein 3  Histone H1.4  Catalase  Neutrophil collagenase  Non-secretory ribonuclease  Leucine-rich alpha-2-glycoprotein  Isoform 1 of Phosphoglucomutase-1  Peptidoglycan recognition protein 1  Matrix metalloproteinase-9  Calponin-2 |  | Cysteine-rich secretory protein 3  Cystatin-SN  Heat shock protein beta-1  Prolactin-inducible protein  Carbonic anhydrase 6  Desmoglein-1  Cystatin-C  Histone H2A type 1  Desmoglein-3  Cystatin-SA  Isoform 1 of Long palate, lung and nasal epithelium carcinoma-associated protein 1  Thioredoxin domain-containing protein 17  Beta-2-microglobulin  Peroxiredoxin-6  Cystatin-S  Isoform 2d of Voltage-dependent L-type calcium channel subunit beta-2  Fatty acid-binding protein 5  Putative uncharacterized protein NCL1 OS=Candida albicans  Cystatin-D  Cornulin  BPI fold-containing family A member 2  cDNA FLJ14473 fis, clone MAMMA1001080, highly similar to Homo sapiens SNC73 protein (SNC73) mRNA  Isoform long of Serine protease inhibitor Kazal-type 5  Isoform 1 of Deleted in malignant brain tumors 1 protein  Highest mean in H as comare to AgP:  Extracellular glycoprotein lacritin  Isoform 1 of Alpha-1-antichymotrypsin  Calmodulin-like protein 5  Keratin, type II cytoskeletal 6A  Isoform 1 of Liver carboxylesterase 1  Interleukin-36 alpha  Antileukoproteinase  Annexin A1  Isoform 1 of Extracellular matrix protein 1  Small proline-rich protein 3  Isoform 1 of Carboxylesterase 2  Cellular retinoic acid-binding protein 2  Histatin-1  Elafin  Alpha-amylase 2B  Cornifin-B  Secretoglobin family 3A member 1 |
| Bostanci et al. (2018) |  |  |  |  | Isoform 1 of Serpin  Isoform 2A of Desmocollin-2  Alpha-2-macroglobulin-like protein 1  Immunoglobulin heavy variable 3-7  Histone H2A type 1  Cornulin  Putative uncharacterized protein BUD2 OS=Candida albicans  Heat shock protein beta-1  Ezrin  Protein FAM3D  Titin, isoform CRA_a  Basic salivary proline-rich protein 2  SPARC-like protein 1  Tigger transposable element-derived protein 3  Isoform 1 of Interleukin-1 receptor antagonist protein  Cystatin-B  Protein-glutamine gamma-glutamyltransferase E  Isoform 1 of WAP four-disulfide core domain protein 2  Mucin-7  Isoform 1 of Long palate, lung and nasal epithelium carcinoma-associated protein 1  Isoform long of Serine protease inhibitor Kazal-type 5  Fatty acid-binding protein 5  Thioredoxin  Leukocyte elastase inhibitor  Cystatin-SN  Desmoglein-1  Carbonic anhydrase 6  Monocyte differentiation antigen CD14  Immunoglobulin lambda-like polypeptide 1  Cystatin-C  Cathepsin B  Cysteine-rich secretory protein 3  Lactotransferrin  Isoform 1 of Deleted in malignant brain tumors 1 protein  Desmoglein-3  Kallikrein-13  IgGFc-binding protein  Cystatin-SA  Prolactin-inducible protein  Alpha-amylase 1  Isoform 2d of Voltage-dependent L-type calcium channel subunit beta-2  cDNA, FLJ93744, highly similar to Homo sapiens keratin 6E (KRT6E), mRNA |
| Bostanci et al. (2018) |  |  |  |  | Isoform 1 of Long palate, lung and nasal epithelium carcinoma-associated protein 1  Isoform long of Serine protease inhibitor Kazal-type 5  Fatty acid-binding protein 5  Thioredoxin  **Leukocyte elastase inhibitor**  **Cystatin**-SN  Desmoglein-1  **Carbonic anhydrase 6**  Monocyte differentiation antigen CD14  Immunoglobulin lambda-like polypeptide 1  **Cystatin**-C  Cathepsin B  Cysteine-rich secretory protein 3  **Lactotransferrin**  Isoform 1 of Deleted in malignant brain tumors 1 protein  Desmoglein-3  **Kallikrein**-13  **IgGFc-binding protein**  **Cystatin**-SA  **Prolactin-inducible protein**  **Alpha-amylase** 1  Isoform 2d of Voltage-dependent L-type calcium channel subunit beta-2  cDNA, FLJ93744, highly similar to Homo sapiens **keratin** 6E (KRT6E), mRNA  Highest mean in H as compared to G:  Calmodulin-like protein 5  Lipocalin-1  Isoform 2d of Voltage-dependent L-type calcium channel subunit beta-2  Cornifin-B  Isoform 1 of Carboxylesterase 2  Cystatin-B  Isoform 1 of Extracellular matrix protein 1  Ig heavy chain V-III region JON  Antileukoproteinase  Isoform 1 of Kallikrein-1 |
| Mertens et al. (2018) | 35 | μg/μl | Hemopexin (HEMO)  α-fibrinogen (FIBA)  Apolipoprotein H (APOH) (only in CP) |  | Plasminogen (PLMN)  Apolipoprotein H (APOH) (only vs CP) |
| Shin et al. (2019) | 744 | pg/mL (ELISA) | Prolactin-inducible protein  Protein S100-A8 (verified with ELISA)  Interferon-induced very large GTPase 1  Protein S100-A9 (verified with ELISA)  Complement C3  Cathelicidin antimicrobial peptide  Myosin-9  Myeloperoxidase  Fibrinogen beta chain  Ig kappa chain V-III region B6  Fibrinogen gamma chain  Phospholipase B-like 1  Transaldolase  Vasodilator-stimulated phosphoprotein  Alpha-actinin-1  Kaliocin-1 (Fragment)  Desmoplakin  Alpha-1B-glycoprotein  Truncated apolipoprotein A-I  Vitronectin  Fibrinogen alpha chain  Neutrophil defensin 3  Galectin-3-binding protein  Actin, aortic smooth muscle  Cystatin-A  Golgin subfamily A member 4  Haptoglobin  Heat shock 70 kDa protein 1A/1B, Isoform 2  POTE ankyrin domain family member I  Programmed cell death 6-interacting protein  Ras-related protein Tab-17  SPARC-like protein 1  Tropomyosin alpha-3 chain, Isoform 4 |  | Zinc-alpha-2-glycoprotein  Carbonic anhydrase 6  Ig mu chain C region  BPI fold-containing family B member 1  Immunoglobulin J chain (Fragment)  Cystatin-B  Kallikrein-1  Lactoperoxidase  Protein S100-A6 (Fragment)  Putative DNA-binding protein inhibitor ID-2B  Alpha-2-macroglobulin-like protein 1  Gelsolin  Beta-2-microglobulin form pI 5.3 (Fragment)  Ig lambda chain V-IV region Hil  Uncharacterized protein  Constitutive coactivator of peroxisome proliferator-activated receptor gamma  Protein disulfide-isomerase  Peptidyl-prolyl cis-trans isomerase A  Fructose-bisphosphate aldolase  Glutamine--fructose-6-phosphate aminotransferase [isomerizing] 2  Peroxiredoxin-1  Hemopexin  78 kDa glucose-regulated protein  Ig lambda chain V-III region SH  Poly [ADP-ribose] polymerase 4  Ig kappa chain V-III region GOL  Cathepsin G  Nucleobindin-2  Phosphoglycerate mutase 1  Sperm flagellar protein 2, Isoform 2 of  Bridging integrator 3  Doublesex- and mab-3-related transcription factor 2  Neutrophil gelatinase-associated lipocalin  Coiled-coil domain-containing protein 175  Protein YIPF6 (Fragment) |
| Tang et al. (2019) | 91 | Da (peaks) | 1044.0 Da  1122.0 Da (segment of immunoglobulin kappa variable 4–1)  1147.1 Da (haptoglobin)  1583.9 Da  3434.4 Da | 1044.0 Da  1122.0 Da (segment of immunoglobulin kappa variable 4–1) | Higher than CP:  1836.4Da  1858.4 Da  Higher than G:  4918.2 Da |
| Antezack et al. (2020) | 217 | Da (peaks) | 3372 Da  3443 Da  3519 Da  3550 Da  6352 Da  6735 Da  12692 Da  13461 Da |  | 2620 Da  7746 Da |
| Hartenbach et al. (2020) | 473 | Log2 transformed peak intensity | Salivary acidic proline-rich phosphoprotein  Submaxillary gland androgen-regulated protein 3B  Cystatin-SA |  | Alpha-2-macroglobulin-like protein  Metalloproteinase inhibitor 1  Apolipoprotein A-I  Transthyretin  Serum albumin  Hemopexin  Catalase  Annexin A1  Protein disulfide-isomerase  Cathepsin G  Keratin, type I cytoskeletal 16  P09972 Fructose-bisphosphate aldolase C  Keratin, type I cytoskeletal 10  Keratin, type I cytoskeletal 13  Matrix metalloproteinase-9  Keratin, type II cytoskeletal 4  Cofilin-1  Calmodulin-like protein 3  Leukocyte elastase inhibitor  Lipocalin-1 (Tear lipocalin)  Protein S100-A11 (Calgizzarin)  Keratin, type I cytoskeletal 9  Rho GDP-dissociation inhibitor 2  Keratin, type II cytoskeletal 2 oral  Protein-glutamine gamma-glutamyltransferase E  BPI fold-containing family B member 1  Cornulin |
| Grant et al. (2022) | 314 | Ratio (periodontitis/health values) | Actin gamma 1  Rho GDP dissociation inhibitor beta  Haemoglobin-beta  Talin-1  Plastin-2  Carbonic anhydrase 1  Profilin 1  S100A12  Keratin 4  Myosin 9  Pyruvate kinase | Actin gamma 1  Rho GDP dissociation inhibitor beta  Haemoglobin-beta  Talin-1  Plastin-2  Carbonic anhydrase 1  Profilin 1  S100A12  Myosin 9  Alpha-1-acid glycoprotein 1  S100A8 | H vs G:  Keratin type II cytoskeletal 4  Pyruvate kinase |
| Grant et al. (2022) |  |  | Alpha-1-acid glycoprotein 1  S100A8  S100A9  MMP9 | S100A9  MMP9 |  |
| Casarin et al. (2023) | 74 | Normalized spectral counts  pg/m | Alpha-amylase 1  Apolipoprotein A-I, Isoform of P02647  Cysteine-rich secretory protein 3, Isoform of P54108  Alpha-enolase  Fibrinogen beta chain, Isoform of P02675  Fibrinogen gamma chain, Isoform of P02679  Hemoglobin subunit alpha  Hemoglobin subunit beta  Heat shock protein beta-1, Isoform of P04792  Ig gamma-1 chain C region, Isoform of P01857  Ig gamma-2 chain C region (Fragment), Isoform of P01859  Ig gamma-4 chain C region (Fragment), Isoform of P01861  Ig mu chain C region (Fragment), Isoform of P01871  Ig lambda-2 chain C regions (Fragment), Isoform of P0CG05  Keratin, type I cytoskeletal 10  Keratin, type II cytoskeletal 1  Keratin, type I cytoskeletal 9  Lactoperoxidase, Isoform of P22079  Profilin-1 (Fragment), Isoform of P07737  Prolactin-inducible protein  Alpha-1-antitrypsin, Isoform of P01009  Submaxillary gland androgen-regulated protein 3B  Vimentin, Isoform of P08670  Zymogen granule protein 16 homolog B |  | Alpha-2-macroglobulin  Actin, cytoplasmic 1  BPI fold-containing family A member 2  Cystatin-S  IgGFc-binding protein, Isoform of Q9Y6R7  Isoform Long of Glucose-6-phosphate 1-dehydrogenase, Isoform of P11413  Glutathione S-transferase P, Isoform of P09211  Hemopexin  Ig alpha-1 chain C region  Ig alpha-2 chain C region (Fragment), Isoform of P01877  Ig alpha-2 chain C region (Fragment), Isoform of P01877  Immunoglobulin J chain  Keratin, type I cytoskeletal 13, Isoform of P13646  Keratin, type II cytoskeletal 4  Lactotransferrin (Fragment), Isoform of P02788  Peptidyl-prolyl cis-trans isomerase, Isof |
| Romano et al. (2023) | 2 | NR |  |  | Cystatin SN (CST1) |

Da, Dalton; ELISA, enzyme-linked immunosorbent essay; nLC-MS/MS, nano-liquid chromatography - tandem mass spectrometry; NR, not reported
