# Supplementary material for: Salivary Proteomics for Detecting Novel Biomarkers of Periodontitis: A Systematic Review
Source: J Periodontal Res. 2024 Dec 2;60(7):633–55. doi: 10.1111/jre.13357 (PMC12371805; doi:10.1111/jre.13357)
Supplement: Supplementary file 4 — Table S3. [file JRE-60-633-s004.docx]

**Supplementary table S3** Analysis of diagnostic accuracy

| Authors | Method of analysis | Main findings |
| --- | --- | --- |
| Bostanci et al. (2018) | Determination of ROC curve and the measurement of AUC for both single proteins and combinations  Logistic regression and machine learning model to obtain a panel of proteins of high predictive value with maximum AUC > 0.97 | Twelve proteins with good predictive power between periodontal diseases and health:  Isoform of serine protease inhibitor Kazal-type 5: AUC = 0.87  Alpha-1-antichymotrypsin: AUC = 0.87  Interleukin-1 receptor antagonist protein: AUC = 0.86  Clusterin: AUC = 0.86  Serpin B3: AUC = 0.85  Leukocyte elastase inhibitor: AUC = 0.85  Deleted in Malignant Brain Tumors 1: AUC = 0.84  Cystatin-SN: AUC = 0.84  Heat shock protein beta-1: AUC = 0.83  Serpin B5: AUC = 0.83  Alpha-1-antitrypsin: AUC = 0.83  Aldo-keto reductase family 1 member B10: AUC = 0.83  Proteins of high predictive value with maximum AUC > 0.97:  MMP-9  Ras-related protein-1  Actin-related protein 2/3 complex subunit 5  (higher expression levels in disease)  Clusterin  Deleted in Malignant Brain Tumors 1  (higher expression levels in health) |
| Tang et al. (2019) | ROC curve analysis of seven peaks which were significantly different between chronic periodontitis and periodontal health | 1044.0: AUC = 0.756 (CI 0.576–0.935)  1122.0: AUC = 0.860 (CI 0.715–1.000)  1147.1: AUC = 0.706 (CI 0.525–0.886)  1583.9: AUC = 0.688 (CI 0.493–0.882)  1836.4: AUC = 0.776 (CI 0.619–0.933)  1858.4: AUC = 0.794 (CI 0.637–0.951)  3434.4: AUC = 0.765 (CI 0.599–0.930) |
| Antezack et al. (2020) | Binary discriminant analysis method followed by a 10-fold cross-validation to build a diagnostic decision tree | Selected peaks:  2620 Da  3372 Da  3519 Da  3550 Da  4139 Da  6735 Da  Sensitivity = 70.3% (± 0.211)  Specificity = 77.8% (± 0.165) |
| Grant et al. (2022) | Determination of ROC curve and AUC for seven candidate biomarkers  Leave-one-out cross-validation approach to determine ROC and AUC for up to four proteins combinations (plus age)  Highest performing combinations to differentiate between health/gingivitis versus periodontititis, health versus gingivitis and mild periodontitis versus advanced periodontitis | Health/gingivitis versus periodontitis:  MMP9 + A1AGP + PK: AUC = 0.954 (CI 0.936–0.972), sensitivity = 81%, specificity = 97%  MMP9 + A1AGP + PK + S100A8: AUC = 0.960 (CI 0.943–0.977), sensitivity = 97%, specificity = 82%  MMP9 + A1AGP + PK + S100A8 + age: AUC = 0.970 (CI 0.956–0.984), sensitivity = 98%, specificity = 37%  [Profilin: AUC = 0.8368 (CI 0.7692 - 0.9044)  S100A8:  AUC = 0.7380 (CI 0.6565 - 0.8196)]*  Health versus gingivitis:  MMP9 + A1AGP + PK: AUC = 0.772 (CI 0.718–0.826), sensitivity = 82%; specificity = 82%  MMP9 + A1AGP + PK + S100A8: AUC = 0.768 (CI 0.713–0.823), sensitivity = 74%, specificity = 79%  MMP9 + A1AGP + PK + S100A8 + age: AUC = 0.764 (CI 0.709–0.819), sensitivity = 74 %, specificity = 79%  Mild periodontitis versus advanced periodontitis:  MMP9 + A1AGP + PK: AUC = 0.768 (CI 0.715–0.821), sensitivity = 71%, specificity = 64%  MMP9 + A1AGP + PK + S100A8: AUC = 0.767 (0.714–0.820), specificity = 34%, sensitivity = 95%  MMP9 + A1AGP + PK + S100A8 + age: AUC = 0.789 (CI 0.738–0.840), specificity = 57%, sensitivity = 80% |

A1AGP: alpha-1-acid glycoprotein AUC, area under the curve; MMP-9: matrix metalloproteinase-9; PK: pyruvate kinase; ROC, receiver-operating characteristic.

*data kindly provided by Authors on request.
